# Validation of the Colombian version of the Karolinska sleepiness scale

**DOI:** 10.5935/1984-0063.20220006

**Published:** 2022

**Authors:** María Camila Laverde-López, Franklin Escobar-Córdoba, Javier Eslava-Schmalbach

**Affiliations:** 1 Universidad Nacional de Colombia, Faculty of Medicine, Department of Internal Medicine, Aerospace Medicine Specialty - Bogotá - Bogotá D.C. - Colombia.; 2 Universidad Nacional de Colombia, Faculty of Medicine, Psychiatry Department - Bogotá - Bogotá D.C. - Colombia.; 3 Fundación Sueño Vigilia Colombiana, Management Office - Bogotá - Bogotá D.C. - Colombia.; 4 Universidad Nacional de Colombia, Hospital Universitario Nacional de Colombia - Bogotá - Bogotá D.C. - Colombia.

**Keywords:** Disorders of Excessive Somnolence, Colombia, Validation Studies, Sleep Wake Disorders (MeSH)

## Abstract

**Introduction:**

Currently, daytime sleepiness is a prevalent condition worldwide. Locally validated instruments for measuring sleepiness are required. The objective of this study was to validate a version of the Karolinska sleepiness scale that was translated into the Spanish spoken in Colombia.

**Methods:**

Individuals who attended a sleep laboratory for a polysomnography study and people in the general population were included. The validation process was performed in 6 phases: translation and back translation of the original version of the scale (English), face validity (n=13), pilot test (n=20), criteria validity (n=139) by means of polysomnography and the Epworth sleepiness scale, reproducibility (n=34), and sensitivity to change (n=40).

**Results:**

Regarding its discriminant validity, the Colombian version of the Karolinska sleepiness scale is correlated with the Epworth sleepiness scale, provided that a Mann-Whitney z=2661 (p=0.0078) was obtained. The scale has an acceptable reproducibility, Spearman Rho=0.55 (p=0.0002), and sensitivity to change, as shown through a two-tailed t test (p=0.0000).

**Conclusions:**

The Karolinska Sleepiness Scale was successfully adapted to the Spanish variation spoken in Colombian and to the conditions of adult Colombians; thus, it constitutes a valid, reliable, and easy to use instrument for the assessment of patients with hypersomnia.

## INTRODUCTION

At present, people sleep 25% less time than 100 years ago and it is estimated that 12 to 20% of people in industrialized countries work on shifts^1^. This situation has caused shift workers to experience sleep-wake cycle alterations associated with voluntary sleep deprivation^1,2^. Excessive daytime sleepiness (EDS) occurs in individuals who show an irresistible desire to sleep under any circumstances^3,4^ and represents a serious threat to society, as there are numerous high-risk activities that require a constant level of alertness and situational awareness when being performed. Therefore, in certain situations, measuring sleepiness is important for protecting the health and quality of life of the general population^3,5,6^.

A person with EDS is defined as someone who expresses an irresistible desire to sleep in any situation, even when engaged in activities demanding a high level of alertness. Before it is considered a disease, EDS must occur most of the day during several weeks or months^7^.

People with EDS show an unavoidable tendency to take naps and to fall asleep in situations that favor sleep, including watching television while sitting down, reading, traveling by car or bus, especially in long and boring trips, talking and/or eating. As a result, they have problems in keeping an adequate state of alertness, and their motor and cognitive activity is decreased as they spend more hours sleeping in a 24-hour period compared to those without EDS^7,8^.

Furthermore, some authors have described EDS as the occurrence of “sleep attacks”, that is, episodes described by patients as “blackouts” in which they cannot resist the compelling feeling of sleepiness and fall sleep suddenly^9^.

EDS is a common problem, and it has been estimated that, in Colombia, about 13.7% (IC95%:12.3-15.3%) of the general population experience it^10^, affecting both their quality of life and the quality of life of the people close to them. There are subjective and objective methods that help diagnose EDS^5,6,11,12^. Usually, self-reporting methods such as the Karolinska Sleepiness Scale (KSS), the Epworth Sleepiness Scale (ESS) and the Stanford Sleepiness Scale (SSS), together with clinical tests such as polysomnography (PSG), actigraphy, the multiple sleep latency test (MSLT) (considered the gold standard for EDS diagnosis), the maintenance of wakefulness test, and 24-hour sleep monitoring studies, are used to reach a diagnosis^3,5,12,13^. However, these specific diagnostic tests are expensive and timeconsuming, so a useful, simple, fast, accessible, and low-cost diagnostic method that can be used in Colombian population is required. For example, the MSLT is performed during the day (at the opposite time to the main sleep period) in order to confirm the presence of EDS and determine its impact on the individual. This test consists of recording brain, eye and muscle electrical activity during four to five naps taken every two hours and with each nap lasting twenty minutes. A sleep latency less than five minutes is considered abnormal and suggestive of hypersomnia^14^. Taking this into account, the objective of this study was to translate the KSS into Spanish so that it can be used in Colombian population, and to evaluate the face validity, internal consistency, criterion validity, discriminant validity, reproducibility and sensitivity to change of the Spanish translation version of the scale in clinical practice.

## MATERIAL AND METHODS

This study was approved by the Research Ethics Committee of the Faculty of Medicine of the Universidad Nacional de Colombia.

### Instrument

The KSS was created under the assumption that certain accidents and disasters are related to the occurrence of sleepiness and that, therefore, it was necessary to establish a method for measuring sleepiness^5^. For decades, it has been known that sleepiness is associated with the alpha and theta activity shown in electroencephalograms (EEG)^15^. In this sense, Akerstedt and Gillberg evaluated the correspondence between electroencephalographic and electrooculographic changes using subjective methods for assessing sleepiness such as the KSS^5^. It should be noted that said study was conducted in eight men whose sleepiness severity had been subjectively rated using the ESS and the visual analogue scale (VAS) before they underwent an EGG or an electrooculogram (EOG) every two hours^5^. Depending on the obtained score, the KSS, a 9-point scale, assesses the level of sleepiness at the time of completing the questionnaire as follows: “extremely alert” (score=1), “alert” (score=3), “neither alert not sleepy” (score=5), “sleepy-but no difficulty remaining awake” (score=7), “extremely sleepyfighting sleep” (score=9). Values between these options (i.e., 2,4,6,8) are considered in the scale but they were not given a verbal equivalent as it happens with 1,3,5,7 and 9^5^. To illustrate the difference in EEG/EOG parameters between high and low sleepiness states, Akerstedt and Gillberg considered maximum (8.6) and minimum (3.1) sleepiness states. In the KSS, the maximum value was found to be very close to the “extremely sleepy-fighting sleep” state, while the lowest value was close to the “alert” state^5^.

Later, several authors added verbal descriptions to each numerical choice of the KSS to avoid biases derived from the fact that individuals might only choose those options that were verbally labeled^16,17,18^. Then, Miley et al. showed that both versions of the KSS (with and without the verbal component) were highly correlated (Kappa=0.73)^19^. In this regard, it has been reported that in the non-verbal version a clear trend towards choosing the numerical options that were verbally labeled is observed, especially options 3, 5 and 7, while in the verbal version this trend is not found^5,20^.

### Phases of the validation process

#### First Phase: Translation and back translation.

First, permission to use and translate the scale to Spanish was obtained from its author. Then, three bilingual persons whose mother tongue was the Spanish spoken used in Colombia independently translated the KSS from its original language (English) into Spanish. These three versions were submitted to a review committee, who was responsible for choosing the version that was most suitable for being used in Colombian population. Said version was evaluated and translated back to English by a second group of translators, native English speakers with a high proficiency and expertise in the Spanish language. Again, this new version was submitted to the review committee, made up of 13 experts, to compare it with the original KSS version, determining that the meaning conveyed in the original instrument had not been lost in the translation and back translation process. The Delphi method was used until consensus was obtained^21^.

#### Second phase: Face validity.

Face validity was performed using the Delphi method, too^21^. In addition, 13 experts in sleep disorders, in two rounds, reviewed and made changes to the Spanish version of the KSS until a 100% consensus was reached.

#### Third phase: Pilot test.

A pilot test was carried out, where the adapted Spanish version of the instrument was administered to 20 adults to determine its administration time and if there were any problems related to the understanding of the scale. The double interview method was used to evaluate adequate understanding of the scale items.

#### Fourth phase: Criterion validity.

**Figure 1:**
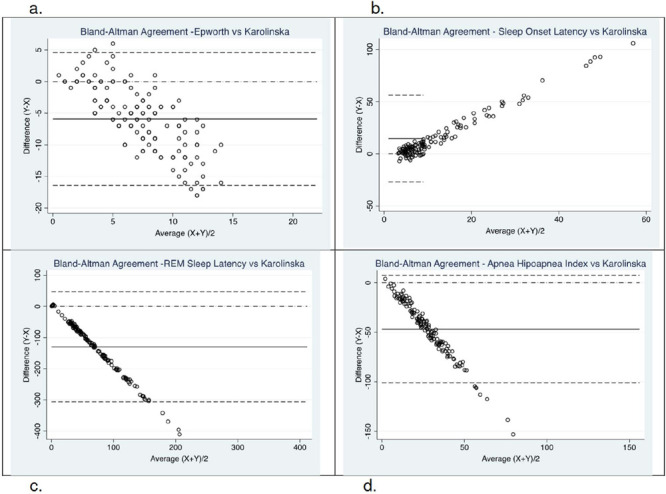
Bland-Altman agreement between Karolinska Sleepiness Scale vs Epworth Sleepiness Scale, Sleep Onset Latency, REM Sleep Latency and Apnea-Hypopnea Index.

Criterion validity was performed by administering the KSS to 139 individuals treated in a sleep clinic. Subjects were asked to complete the questionnaire right before the lights were turned off to perform the PSG, so that it was possible to compare the score obtained in the KSS with the sleep onset latency (SOL) and other variables. Bland-Altman agreement was used to compare KSS score with SOL, REM sleep latency (RSL), and the apnea-hypopnea index (AHI) (Figure 1). Also, the Mann Whitney U test was used to compare KSS scores with ESS scores (>10 vs <=10); SOL (<=10min vs SOL>10min); RSL (<=120min vs >120min); and AHI (>30 events/hour vs <=30 events/hour). Additionally, an extreme values validation of the KSS was done, comparing them with ESS, SOL, RSL and AHI median values, using the Mann- Whitney U test.

Note:

a. Epworth Sleepiness Scale vs Karolinska Sleepiness Scale: Lin's Concordance Correlation coeff, =-0.0214.

b. Sleep Onset Latency vs. Karolinska Sleepiness Scale. Lin's Concordance Correlation coeff =0.0924.

c. REM Sleep Latency vs Karolinska Sleepiness Scale: Lin's Concordance Correlation coeff= 0.0003.

d. Apnea-Hypopnea Index vs Karolinska Sleepiness Scale: Lin's Concordance Correlation coeff=0.0030.

#### Fifth phase: Reproducibility.

Reproducibility was measured by administering the scale to 34 medical students. To remove the effect of memory in the participants’ responses, eight days after the first administration, they were asked to complete the instrument again under similar conditions of time, activity, and place in order to assess similarity between responses. The Spearman rho was used to test the correlation in the KSS results between both moments.

#### Sixth phase: Sensitivity to change.

Sensitivity to change was assessed by asking 40 individuals, on the one hand, to complete the KSS right before going to sleep and, on the other, to complete it again upon waking up the next morning; in addition, they were asked to evaluate their sleep quality during that night using the VAS. A sleep quality >6 cm according to the VAS was established as an acceptable indicator. Then the initial values of the KSS before and after sleeping were compared, considering an appropriate sleep quality as the intervention for sleepiness.

## RESULTS

### Translation - back translation

A comparative analysis of the three translated versions of the KSS was carried out to assess their consistency and to analyze any grammatical discrepancies between them, finding that there were no marked differences in any version regarding the translation of the question and the possible answer options, except for answer options No. 4 and No. 9.

In the original version of the scale, “Rather alert” is used for answer options No. 4 and No. 9. In the original version of the scale, “Rather alert” is used for option No. 4, which was translated in each version as “más bien alerta” (rather alert), “algo alerta” (somewhat alert), and “más o menos alerta” (more or less alert). Both, researchers and translators were asked to choose by consensus the best translation option, taking into account the logical order of severity of the scale and the type of language commonly used in Colombia, and finally “más o menos alerta” (more or less alert) was chosen. In the case of answer option No. 9 “fighting sleep”, it was also translated differently in the three versions: “luchando contra el sueño” (fighting sleep), “peleando con el sueño” (struggling with sleep) and “combatiendo el sueño” (battling sleep). Using a similar methodology, both, researchers and translators decided that “luchando contra el sueño” (fighting sleep) was the most appropriate term to use.

Subsequently, the scale was back translated based on the Delphi method^21^. The back translated versions were similar, except for items No. 4 and No. 9. In the case of answer option No. 4 “más o menos alerta”, 2 different terms were used: 2 translators chose using “more or less alert”, while the third one chose “somewhat alert”. Once again, through consensus it was decided that “more or less alert” was the most appropriate term, since, despite it did not exactly match the term used in the original version of the scale (rather alert), it retained the original meaning. Regarding item No. 9, a discrepancy was found in the terms used for translating “gran esfuerzo para mantenerse despierto” (having trouble staying awake), since the following options were provided by the three translators: “tried very hard to stay awake”, “having trouble staying awake” and “great difficulty to keep awake”. In this case it was considered that, despite the obvious differences between these options, all of them kept the original meaning of the sentence, as well as the original response intention, so the three of them were considered appropriate.

### Face validity

A total of 13 experts in sleep disorders participated in the face validity process. In the first round, all experts considered the Colombian version of the scale was relevant to assess the level of sleepiness. In addition, 46% of them agreed the items of the scale were appropriate and approved its use, since they considered it to be simple, clear and easy to understand by the general population. 4 experts deemed necessary to replace the term “nivel de sueño” (level of sleep) by “somnolencia” (sleepiness), the closest term to the one used in the original version of the scale, because it was more faithful to the original version and the purpose of the scale was to assess the level of sleepiness. One of the experts proposed changing the word “alerta” (alert) to “despierto” (awake) on the basis that the latter was more commonly used in Colombia when talking about sleep level, and that “alerta” (alert), according to him, was generally used in a military context and could lead to confusion. Another expert stated that the original version of the scale imposes a response bias to the respondent by presenting verbal descriptions for the odd-numbered items, thus leading them to choose these response choices, so he proposed validating the KSS version with a verbal component, in which new information is included in the scale question and verbal descriptions are added for the remaining response items^20^. In this regard, Miley et al, reported a high correlation between the original KSS and the current version, that is, the one with a verbal component^19^. Considering this information, and following this suggestion, the KSS version with the verbal component was validated to avoid said response bias. After following the suggestions proposed by the experts, a second experts’ round was conducted (Delphi method), achieving a 100% agreement among all experts regarding relevance, simplicity, clarity, and ease of use of the KSS.

### Pilot test

The average response time was 34.5 seconds (range: 20-53). When the scale was administered, seven individuals did not understand the instruction regarding how to assess the level of sleepiness experienced during the five minutes prior to answering the questionnaire. So, a new instruction was added to make it more clear for respondents: “circle the number that represents your level of sleepiness during the five minutes immediately prior to this test”.

### Criteria validity

The KSS was administered to 139 adults (18-90 years old), of which 41% (n=57) were men and 59% (n=82), women. Polysomnographic data obtained during the administration of both scales, the Colombian version of the KSS and the ESS, were analyzed: SOL, RSL, AHI, the periodic leg movement index (PLMI), and the arousal index (AI) (Table 1). Since age was the only variable in which normal distribution was observed, mean and standard deviations were used for describing it. For the description of the remaining variables, median and interquartile range measurements were used. The analysis of the PLMI is not shown in Table 1 due to the low number of pathological scores and the resulting statistical limitation (seven subjects with a pathological score, PLMI>5/hour).

**Table 1:** Polysomnographic characteristics and scales used in the study population.

Age	n=139 (n)	SOL (Med, IQR)	RSL (Med, IQR)	AHI (Med, IQR)	ESS (Med, IQR)	KSS (Med, IQR)
0-39	Women (11)	12.5 (7-31))	87.5 (61.5-232.5)	21.3 (8.9-22.2)	9 (6-16)	4 (2-5)
	Men (12)	12 (7.25-20.25)	133.5 (94.125- 174.375)	57.2 (19.025-85.1)	11 (6.75-14.75)	4 (3-7)
40-59	Women (39)	12.5 (6.5-22.5)	147 (82.5-191)	42.2 (26-57.7)	10 (4-13)	4 (3-6)
	Men (27)	8.5 (6.5-14.5)	76 (56-161.5)	56.3 (40.7-70.9)	12 (8-17)	4 (3-6)
60 +	Women (32)	10.5 (6.125-21.375)	123.5 (75.125-238.5)	54.85 (41.725-67.7)	9 (6.25-15))	5 (3-6)
	Men (18)	12.5 (8-40.5)	91.25 (39-166)	55.9 (39.55-75.675)	9.5 (4-13)	4 (2-5.25)

X: mean; SD: standard deviation; KSS: Karolinska sleepiness scale; ESS: Epworth sleepiness scale; SOL: sleep onset latency (in minutes); RSL: REM sleep latency (in minutes); AHI: apnea-hypopnea index; Med: median; IQR: interquartile range.

A Spearman’s rank correlation test was performed for each variable mentioned above to compare their respective values with the results obtained in the Colombian version of the KSS, finding that the values of the latter are independent, showing the following Spearman’s Rho values: ESS=0.29, SOL=-0.15, RSL=0.01, AHI=0.08 and AI=0.06.

Then, a correlation analysis between the values obtained for the Colombian version of the KSS and the groups considered as pathological for each one of the variables was performed. In the case of ESS, a score of 10 was established as the cut-off point, for, according to that instrument, it means high levels of daytime sleepiness. The median score in the KSS for subjects who had a pathological score in the ESS (>10) was different from those who had a score ≤10 points, with a Mann-Whitney value=-2.661 and Prob>|z|=0.0078, which were statistically significant results. No statistically significant correlation was found for the remaining variables that were evaluated (Table 2).

**Table 2:** Criteria validity by means of the correlation of the Colombian version of the Karolinska sleepiness scale.

		n=139 n (%)	KSS (Med, IQR)	p			
**ESS**	>10	n = 73 (53%)	4 (2-5.5)	0.0078*			
	≤10	n = 66 (47%)	4 (3-6.3)				
**SOL**	≤10 min	n=69 (49%)	4 (3-6)	0.4401			
	>10 min	n=70 (51%)	4 (3-6)				
**RSL**				≤120 min	n=66 (47%)	4 (3-6)	0.8011
				>120 min	n=66 (47%)	4 (3-6)	
**AHI**	>30/h	n=105 (76%)	4 (3-6)	0.6401			
	≤30/h	n=34 (24%)	4 (3-6)				

*Mann-Whitney (z=-2.661); KSS: Karolinska sleepiness scale; ESS: Epworth sleepiness scale; SOL: sleep onset latency (in minutes); RSL: REM sleep latency (in minutes); AHI: apnea-hypopnea index; Med: median; IQR: interquartile range.

Discriminant validity was assessed using the maximum extreme values of the Colombian version of the KSS, in which response options 7, 8 and 9 were considered as the maximum extreme values for sleepiness level (Table 3). A statistically significant difference was found between the median values of the adapted version of the KSS and those of the ESS (Mann-Whitney=-2.084, Prob>|z|=0.0371). On the contrary, no statistically significant differences were found in relation to the other variables.

**Table 3:** Correlation of the maximum extreme values of the Karolinska sleepiness scale.

	KSS≥7 n=22 (16%)	KSS<7 n=117 (84%)	p
**ESS** (Med, IQR)	13 (9-16)	9 (6-13.5)	0.0371*
**SOL** (Med, IQR)	8.3 (4.8-18.6)	115 (7-24)	0.0788
**RSL** (Med, IQR)	131 (73.7-252.2)	112.5 (69-177.2)	0.1276
**AHI** (Med, IQR)	53 (28-79.8)	46.4 (31.6-65.3)	0.2642

*Mann-Whitney (z=-2.084); KSS: Karolinska sleepiness scale; ESS: Epworth sleepiness scale; SOL: sleep onset latency (in minutes); RSL: REM sleep latency (in minutes); AHI: apnea-hypopnea index; Med: median; IQR: interquartile range.

A discriminant validity analysis was performed using the minimum extreme values of the Colombian version of the scale, where response options No. 1 and No. 2 were considered as minimum extreme values of alertness when evaluating the individuals’ level of sleepiness. A statistically significant correlation was found with the ESS values (Mann- Whitney=-4.017, Prob>|z|=0.0001). In addition, a correlation trend was also observed with SOL, but it was not statistically significant (Table 4).

**Table 4:** Correlation of the minimum extreme values of the Karolinska sleepiness scale.

	KSS≤2 n=24 (17%)	KSS>2 n=115 (83%)	P
**ESS** (Med, IQR)	6 (2-9)	11 (7-16)	0.0001*
**SOL** (Med, IQR)	17 (8.1-38.7)	10 (6.5-20.5)	0.0383
**RSL** (Med, IQR)	130.2 (69-222.8)	112.5 (69.5-178)	0.6843
**AHI** (Med, IQR)	46.3 (24.6-58.7)	48 (31.4-66.5)	0.4353

*Mann-Whitney (z=-4.017); KSS: Karolinska sleepiness scale; ESS: Epworth sleepiness scale; SOL: sleep onset latency (in minutes); RSL: REM sleep latency (in minutes) AHI: apnea-hypopnea index; Med: median; IQR: interquartile range.

### Reproducibility

To measure its reproducibility, 34 individuals were administered the Colombian version of the KSS two times 8 days apart, both under similar conditions. Since the results of these 2 measurements did not show a normal distribution, non-parametric tests were performed, obtaining a Spearman’s Rho=0.55 (p=0.0002).

### Sensitivity to change

**Figure 2:**
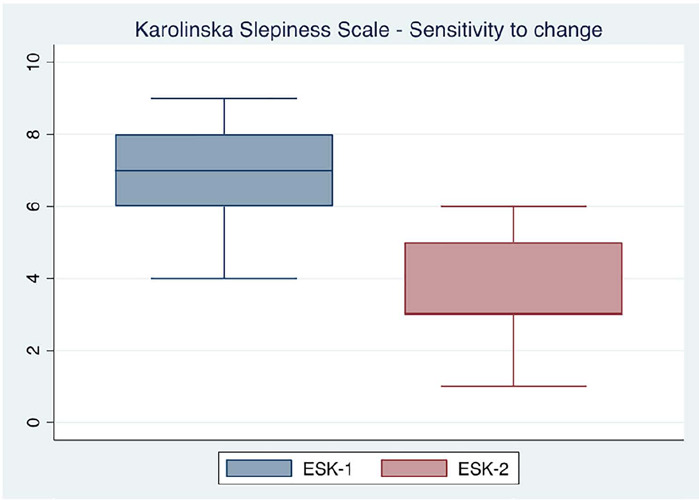
Sensitivity to change of the Karolinska Sleepiness Scale Colombian Version (CV).

Note: ESK-1: Karolinska Sleepiness Scale Colombian Version pre-sleeping; ESK-2: Karolinska Sleepiness Scale Colombian Version post-sleeping; Sign test, p<0.0001.

For assessing the sensitivity to change of the Colombian version of the KSS, 40 individuals were asked to complete the instrument before and after sleeping, and the data obtained in both moments were evaluated. These data did not have a normal distribution, so non-parametric tests were used for their analysis. The median value before sleeping was 7 (IQR=6-8) and after sleeping, post-intervention, was 3 (IQR=3-5), showing a good sleep quality (Sign test, p=0.0000) (Figure 2)

## DISCUSSION

Due to the existing cultural and language differences among countries, the validation of scales is necessary. In the present study, after performing a translation and back translation of the KSS, submitting the translated version to 13 experts for being reviewed, achieving a 100% consensus^21^, and conducting a pilot test, a simple to use and easy to understand adapted version of the KSS for Colombian population was obtained. In addition, since this adapted version preserves the meaning of the original version, it constitutes a qualified instrument that can be completed fast and easy by Colombian individuals with sleepiness symptoms or with a risk of developing it. The results of the present validation study show that there is a similarity between the KSS^5^ and the ESS^3^, the latter being considered an effective test for the diagnosis of sleepiness, thus proving the discriminant validity of the scale and its diagnostic capacity.

The KSS allows assessing sleepiness at a given time, and it has several advantages when used in people who perform high-risk activities or have jobs that require them to have a high level of alertness^15,18,19,20^. According to the analysis of the extreme values of the scale, when choosing the maximum extreme values for classifying the level of sleepiness (i.e., options 7, 8 9), the respondent has a high probability of being experiencing sleepiness symptomatology and, this way, preventive measures can be taken to prevent the occurrence of mistakes or situations threatening the safety of the activities being performed. Adequate values were also found regarding reproducibility, which makes the version of the KSS proposed here a reliable and valuable tool for monitoring sleepiness. In addition, the scale has an appropriate sensitivity to change, which means it can be used as a diagnostic aid tool and as a useful test for measuring any response to sleepiness treatment and any change over time in this population.

The evaluation of a patient with sleep problems begins with a careful clinical assessment that includes a detailed review of their sleep disorders history, their medical record and their psychiatric history, their drug use history, as well as their social and family history. Physical examination should include a general medical examination paying special attention to the upper airway, and a neurological examin^22^. In addition, appropriate objective testing using a polysomnography and a multiple sleep latency test (if needed) will help confirm the diagnosis and guide an adequate treatment plan^22^.

In Colombia, EDS can be assessed using the ESS, which has already been validated in Colombian population^3^, and the MSLT, which is the gold standard for evaluating sleepiness; however, this test is not frequently available in our country, which is why the implementation of easy and fast to use instruments that allow evaluating EDS in Colombian population is required, being one of them the KSS^5^. To the best of our knowledge, so far there is no a validated version of the KSS^5^ that has been translated into the Spanish spoken in Colombia, which allowed the development of this study. Also, it should be noted that the SSS^23,24^ has not yet been validated in Colombian population. Having access to easy to use instruments that allow assessing EDS such as the ESS is of great importance in a country with limited resources like Colombia. In this regard, the ESS is a self-administered questionnaire in which patients are asked to rate the probability of falling asleep in eight specific situations of daily life. Each situation is rated using a 0 to 3 scale, thus the overall score ranges from 0 to 24 points; the higher the score, the higher the degree of daytime sleepiness. According to this scale, the risk of sleepiness is low if the score is less than 11 points, but patients with scores between 11 and 24 points, there is a high risk of sleepiness^25^. However, when compared with the Colombian version of the KSS, the latter offers a faster, simpler and easier administration and allows a similar assessment of EDS.

Finally, after the validation process described here was completed, the Colombian version of the KSS was obtained, which makes available a new inexpensive, accessible, fast, and easily applicable tool for the assessment of sleepiness in the country.

## CONCLUSION

Validating scales allows obtaining measurement instruments that can be used regardless of the nationality or language spoken by the target population. The Colombian version of the KSS presented here was obtained after performing the procedures required for the validation of any instrument, finding that it has an appropriate criteria validity, reproducibility and sensitivity to change, thus making available a low-cost, accessible and easy to use tool for assessing sleepiness in Colombia.
